# G1 Domain of Versican Regulates Hyaluronan Organization and the Phenotype of Cultured Human Dermal Fibroblasts

**DOI:** 10.1369/0022155416643913

**Published:** 2016-04-28

**Authors:** Mervyn J. Merrilees, Ning Zuo, Stephen P. Evanko, Anthony J. Day, Thomas N. Wight

**Affiliations:** Department of Anatomy and Medical Imaging, School of Medical Sciences, University of Auckland, Auckland, New Zealand (MJM,NZ); Matrix Biology Program, Benaroya Research Institute, Seattle, Washington (SPE,TNW); Wellcome Trust Centre for Cell-Matrix Research, The Faculty of Life Sciences, University of Manchester, Manchester, United Kingdom (AJD)

**Keywords:** cables, differentiation, G1 domain, hyaluronan, versican

## Abstract

Variants of versican have wide-ranging effects on cell and tissue phenotype, impacting proliferation, adhesion, pericellular matrix composition, and elastogenesis. The G1 domain of versican, which contains two Link modules that bind to hyaluronan (HA), may be central to these effects. Recombinant human G1 (rhG1) with an N-terminal 8 amino acid histidine (*His*) tag, produced in *Nicotiana benthamiana*, was applied to cultures of dermal fibroblasts, and effects on proliferation and pericellular HA organization determined. rhG1 located to individual strands of cell surface HA which aggregated into structures resembling HA cables. On both individual and aggregated strands, the spacing of attached rhG1 was similar (~120 nm), suggesting interaction between rhG1 molecules. Endogenous V0/V1, present on HA between attached rhG1, did not prevent cable formation, while treatment with V0/V1 alone, which also bound to HA, did not induce cables. A single treatment with rhG1 suppressed cell proliferation for an extended period. Treating cells for 4 weeks with rhG1 resulted in condensed layers of elongated, differentiated α actin-positive fibroblasts, with rhG1 localized to cell surfaces, and a compact extracellular matrix including both collagen and elastin. These results demonstrate that the G1 domain of versican can regulate the organization of pericellular HA and affect phenotype.

## Introduction

The domain structure of the gene and core protein of the extracellular matrix (ECM) proteoglycan versican, through alternative splicing of exons, gives rise to multiple isoforms of different sizes. The largest variant (V0) contains two glycosaminoglycan (GAG) binding regions (α and β), V1 has the β GAG exon only, V2 the α GAG exon only, and the smallest variant V3, with neither GAG exon, is formed from the amino-terminal globular domain (G1) and the carboxyl-terminal domain (G3);^1–3^ a V4 variant mRNA predicting a truncated β GAG domain has recently been identified in human breast cancer.^4^ These transcripts give rise to modular core proteins of different composition and size. The modular nature of the versican core protein creates a highly diverse molecular constituent of the ECM capable of binding to a variety of factors involved in ECM remodeling and regulation of cell phenotype. These variants have different effects on the ECM impacting such events as cell proliferation, cell adhesion and migration, pericellular coat formation, and elastogenesis.^5–11^ However, it is not clear whether specific domains of the core protein of versican can mimic these effects or alternatively might display different biological activities.

The G1 domain at the amino-terminal end of versican, common to all isoforms, is composed of an immunoglobulin-like domain and two contiguous Link modules. In this study, we have investigated the effect of histidine (*His*)-tagged recombinant human G1 (rhG1), produced in the tobacco plant, on the phenotype of cultured human dermal fibroblasts. We report that the G1 domain of versican interacts with endogenously produced hyaluronan (HA) at periodic sites along the HA strand and promotes aggregation of HA strands into cable-like structures in the pericellular matrix (PCM) of the cultured cells. Furthermore, the formation of aggregates of HA in the PCM of the dermal fibroblasts is accompanied by reduced proliferation of these cells. In long-term cultures, G1 induces a differentiated, layered tissue of elongated cells and a compact ECM of collagen and elastin. These studies demonstrate that a specific structural domain within the versican core protein has biological activity and can impact ECM remodeling and cell phenotype.

## Materials and Method

### Recombinant G1

**Figure 1.**
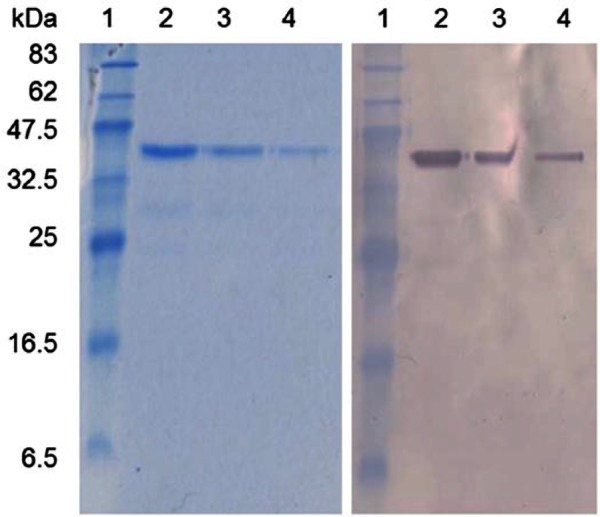
Recombinant human versican V3 (recombinant human G1 [rhG1]), 37 kDa (aa21-346 of P13611 CSPG2_HUMAN) with N-terminal 8 amino acid histidine tag, expressed in *Nicotiana benthamina*. Left panel: Coomassie blue stained sodium dodecyl sulfate polyacrylamide gel electrophoresis (SDS-PAGE). Right panel: Western blot stained with mouse polyclonal to versican. Lane 1: molecular weight marker (kDa); lanes 2 to 4: 0.5, 0.25, 0.1 µg rhG1, respectively.

Recombinant versican G1 domain (aa21-346 of P13611 CSPG2_Human), expressed in *Nicotiana benthamiana*, was produced under contract by Agrenvec, Madrid, Spain. The 37 kDa product was purified by immobilized metal ion chromatography and size exclusion, with protein detection by sodium dodecyl sulfate polyacrylamide gel electrophoresis (SDS-PAGE) and Western blot using versican antibody (H00001462-B01P Abnova, Taipei, Taiwan; see Fig. 1). The expressed sequence was as follows: HHHHHHHHLHKVKVGKSPPVRGSLSGKVSLPCHFSTMPTLPPSYNTSEFLRIKWSKIEVDKNGKDLKETTVLVAQNGNIKIGQDYKGRVSVPTHPEAVGDASLTVVKLLASDAGLYRCDVMYGIEDTQDTVSLTVDGVVFHYRAATSRYTLNFEAAQKACLDVGAVIATPEQLFAAYEDGFEQCDAGWLADQTVRYPIRAPRVGCYGDKMGKAGVRTYGFRSPQETYDVYCYVDHLDGDVFHLTVPSKFTFEEAAKECENQAARLATVGELQAAWRNGFDQCDYGWLSDASVRHPVTVARAQCGGGLLGVRTLYRFENQTGFPPPDSRFDAYCF.

### Preparation of Versican

Versican was purified from bovine aorta by a combination of ion exchange and size exclusion chromatography^7,14^ and was biotinylated using NHS-LC-biotin (Pierce, now Thermo Fisher Scientific, Waltham, MA) according to the manufacturer’s instructions. The preparation consisted of full length V0/V1, with the majority (~90%) being V1 as assessed by Coomassie blue staining of chondroitinase ABC-treated core proteins (data not shown). Biotinylation was done in the presence of HA to minimize potential interference with the HA-binding site. Purified versican (10 mg) dissolved in 5 ml of 0.1 M HEPES, 0.1 M sodium acetate, pH 7.3, was mixed with 500 µg HA (235 kDa average molecular weight, Genzyme, Boston, MA) and incubated for 30 min. NHS-LC-Biotin (1 mg) was then added for 2 hr, followed by dialysis into 4 M guanidine hydrochloride (GuHCL). The bVersican was then purified over HA-Sepharose as described previously.^12,15^

### Cell Culture

Human neonatal dermal fibroblasts from ATCC Primary Cell Solutions (Manassas, VA) were cultured according to protocols provided (ATCC PCS-201-010). Cells were cultured in Dulbecco’s modified Eagle’s medium (DMEM)—high glucose (Invitrogen Cat. No. 10569-044, Carlsbad, CA) supplemented with 10% fetal bovine serum (FBS; Thermo Hyclone Cat. No. SH3046.02, Logan, UT) and glutamine-penicillin-streptomycin (Invitrogen Cat. No. 10378-016). Cells between passages 3 and 7 were used for experiments. Cells were cultured on both plastic and glass coverslips for varying periods up to 4 weeks (times specified in text and figure captions). Selected cultures were treated with 10 µg/ml of *His* tagged-rhG1 (rhG1; empirically determined by dose response of 1, 2, 5, and 10 µg/ml, and by previous studies on versican);^12^ 4 µg/ml of biotinylated HA binding protein (bHABP), composed of aggrecan HA-binding region and cartilage link protein, isolated from bovine nasal cartilage;^13^ and 2, 5, 10, and 15 µg/ml of versican and 10 µg/ml biotinylated versican (bVersican) isolated from bovine aorta.^7,14^ For all treatments, medium was changed when rhG1 and versican were added. In extended cultures out to 4 weeks, medium was changed every 3 days and fresh rhG1 was added. The growth of control and rhG1-treated cells (10 µg/ml) over a 15-day period, with fresh media added on days 2 and 6, reducing rhG1 concentration to 7 and 5.6 µg/ml, respectively, was measured by counting of cells from micrographs of duplicate cultures for each time point (day 0, 9 hr, days 1, 2, 6, 8, 14, and 15) on coverslips in 24-well plates. Photographs were taken on a Nikon Eclipse E400 under a 10× objective lens.

### Immunocytochemistry

Cultures for analysis of cell surface HA and effects of treatments were fixed for 30 min in cold (−20C) 100% methanol. For analysis of treatment with rhG1, fixed cells were washed in phosphate-buffered saline (PBS) 3 × 5 min, blocked with 0.1% donkey serum for 1 hr, and incubated overnight at 4C with bHABP (4 µg/ml) and anti-*His* antibody (Sigma-Aldrich Cat. No. H1029, St. Louis, MO) at 1:100. Cells were washed 2 × 5 min in PBS, and incubated for 1 hr with Streptavidin 488 (Jackson ImmunoResearch Cat. No. 016540084, West Grove, PA) at 1:200 and Alexa 594 goat-anti-mouse IgG (Jackson ImmunoResearch Cat. No. 115545003) at 1:500. Following rinsing in PBS, cells were mounted with ProLong Gold Antifade Mountant with DAPI (Molecular Probes Cat. No. P36935, Eugene, OR). For cultures treated with bHABP and with bVersican, fixed cells were incubated for 1 hr with Streptavidin 488 followed by washing in PBS and mounting. For cultures treated with versican, fixed cells were incubated overnight at 4C with bHABP and antiversican (Abcam Cat. No. ab177480, Cambridge, UK) at 1:100, washed 2 × 5 min in PBS, and incubated for 1 hr with Streptavidin 488 at 1:200 and Alexa 594 goat-anti-mouse IgG at 1:500 followed by washing in PBS and mounting.^13^

### Imaging

Cultured and immunostained cells were imaged on a Nikon Eclipse E400. Morphometric parameters of cables and rhG1 deposits on HA strands were determined from screen images using Adobe Photoshop measurement tools. Four-week multilayered fibroblast cultures were fixed in 4% paraformaldehyde for 30 min, and samples processed for paraffin embedding and sectioning and for electron microscopy. For the latter, tissue samples were postfixed in 2.5% glutaraldehyde. Ultrathin sections, stained with uranyl acetate, lead citrate, and tannic acid, were viewed on a Tecnai G^2^ Spirit Twin transmission electron microscope.

## Results

The 37 kDa recombinant G1 domain from human versican was purified by immobilized metal ion and size exclusion chromatography, with protein detection by SDS-PAGE and Western blot using a polyclonal versican antibody (Fig. 1).

**Figure 2.**
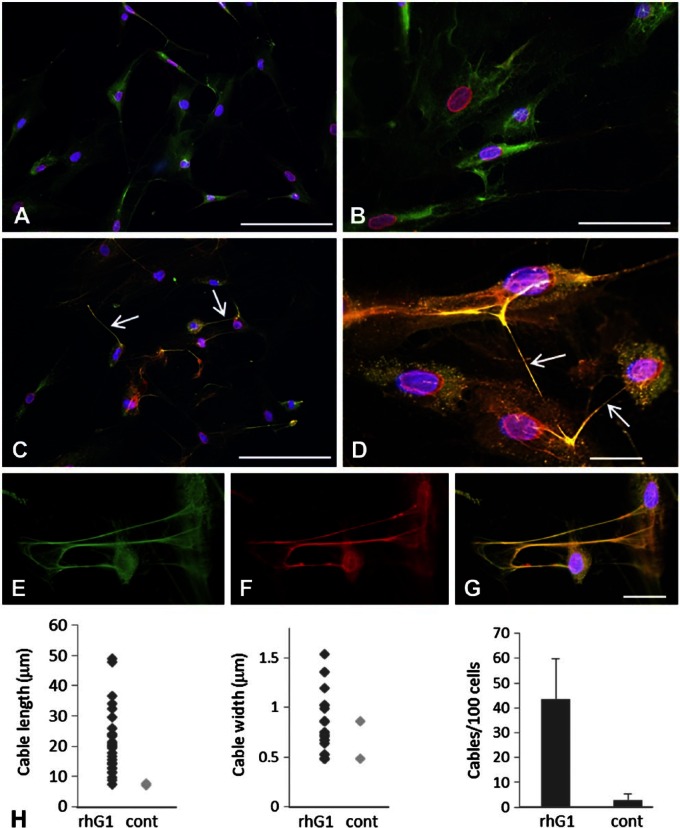
Control (A, B) and recombinant human G1 (rhG1) treated (10 mg/ml) (C, D) cultured human dermal fibroblasts, stained with biotinylated hyaluronan binding protein (bHABP)/streptavidin (green) to detect HA and with antibody to histidine (*His*) tag on G1 (red). rhG1-treated cultures show colocalization of rhG1 with hyaluronan (yellow) and induction of cable structures often extending between cells (arrows). Cables stained with bHABP/streptavidin (E), antibody to *His* tag on G1 (F), and merged images (G). Distribution of cable lengths, widths, and abundance in the presence or absence of rhG1 (H). Scale bars A, C, 50 µm; B, 25 µm; D, E, F, G, 10 µm.

Treatment of cultured low-density dermal fibroblasts for 24 hr with 10 µg/ml of rhG1 induced formation of HA cable-like structures extending up to 50 µm from and between cells (Fig. 2A–D). Staining of HA with bHABP/streptavidin and with an antibody to the *His* tag of rhG1 showed localization of G1 to the HA cables (Fig. 2E–G). Mean cable lengths and widths (±SEM) were 21.3 ± 2.0 and 0.8 ± 0.3 µm, respectively, with ~40% of cells associated with cables (Fig. 2H). Control cultures had very few cables, which were short and did not extend between cells.

**Figure 3.**
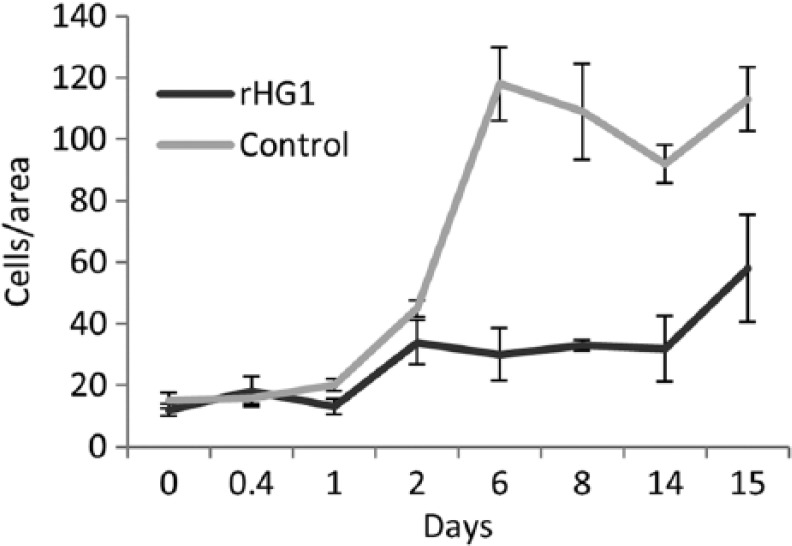
Effect of single dose of recombinant human G1 (rhG1; 10 mg/ml) at day 0 on cell growth over 15 days. Error bars SEM of triplicate cultures.

Extended culture of rhG1-treated cells out to 15 days, with addition of fresh media at days 2 and 6 (without fresh rhG1), showed that a single dose of rhG1 slowed growth significantly throughout the culture period compared with untreated controls (Fig. 3).

**Figure 4.**
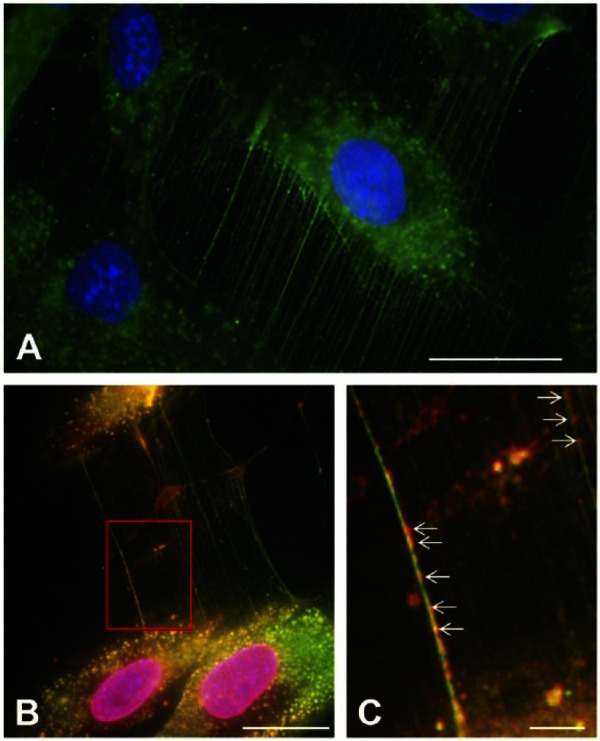
A. Strands of hyaluronan (HA), stained with biotinylated HA binding protein (bHABP)/streptavidin (green) extending between cultured dermal fibroblasts. Nuclei stained with DAPI. B. Dermal fibroblasts exposed to 10 mg/ml recombinant human G1 (rhG1) for 24 hr and stained with bHABP/streptavidin and antibody to histidine (*His*) tag (red) on rhG1. C. Boxed area in B enlarged to show periodic binding of rhG1 *His* tag to HA strands (arrows). Scale bars A, B, 10 µm; C, 2 µm.

Cultures of control dermal fibroblasts, at low density and stained with bHABP/streptavidin (Fig. 4A), showed multiple HA strands of variable thickness and brightness, extending from the cell surfaces with many of the strands bridging between adjacent cells. In cell cultures treated with rhG1 (10 µg/ml) for 24 hr (fixed, double stained with HABP/streptavidin and anti-*His*, and viewed by oil immersion) periodic *His* staining (Fig. 4B) was evident, both on individual HA strands and on coalesced strands, the latter usually at the edge of multiple strand bundles. rhG1 stained with *His* antibody showed a variable periodicity, and on single HA strands, no overlap with bHABP staining was seen (Fig. 4C). Where HA strands were thicker (aggregated), some overlap of the red and green staining was evident, but distinct *His* staining was still clearly discernible (Fig. 4C).

**Figure 5.**
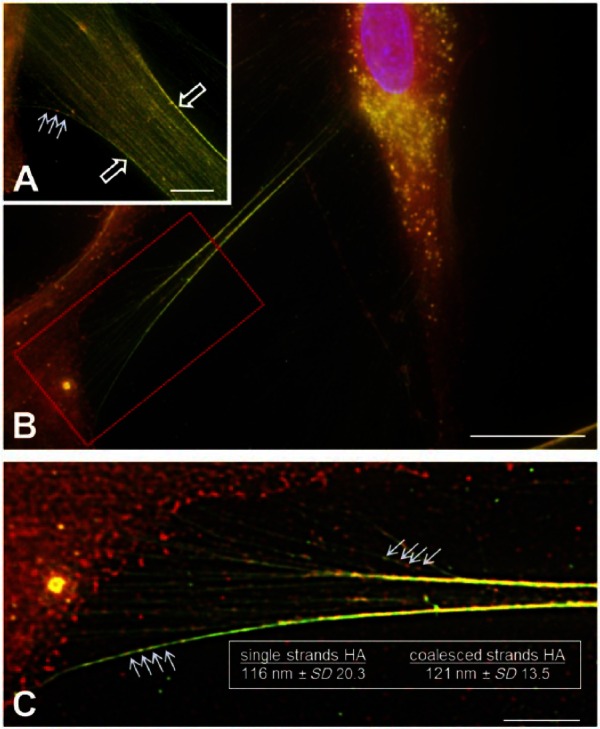
Coalescence of hyaluronan (HA) strands and cable formation induced by recombinant human G1 (rhG1). A. Coalescence of strands (open arrows), rhG1 on HA strands (red/yellow) indicated by closed arrows. B. Cable extending between two cells with peripheral aggregation of HA strands. C. Boxed area in B showing coalescence of individual HA strands arising from cell surface to form a cable. Spacing of rhG1 (closed arrows) is similar on individual (top group of arrows) and on coalesced strands (lower group of arrows) indicating alignment of rhG1 molecules. Scale bars A, C, 1 µm; B, 10 µm.

The aggregation of HA strands into cables was best seen as strands were progressively drawn together at increasing distance from the cell surface (Fig. 5). Close to the cell surface, individual strands were clearly visible, but with increasing distance from the cell surface, the aggregation of strands progressively increased with multiple strands drawn into cables in which the colocalization of HABP/streptavidin and anti-*His* staining produced merged yellow fluorescence (Fig. 5A, B), again prominent on the edges of cables. Close to the cell surface, where both individual and coalesced strands were resolvable with digital magnification (Fig. 5C), the periodic *His* staining could be measured. The periodicity on aggregated and individual strands was not significantly different (121 nm ± *SD* 13, *n* = 55; 116 ± 20 nm, *n* = 58), interpreted as rhG1 molecules or clusters of rhG1 remaining in register following aggregation of HA strands. The rhG1-stained deposits on the merged HA strands were significantly (*p* < 0.001) larger than on single strands (diameter on single strands 31.2 ± *SD* 5.3 nm; two strands 45.9 ± 5.0 nm; three or more strands 46.9 ± 11.6 nm), suggesting that the rhG1 molecules may interact to form homotypic clusters, similar to those reported by Murasawa et al.^16^

**Figure 6.**
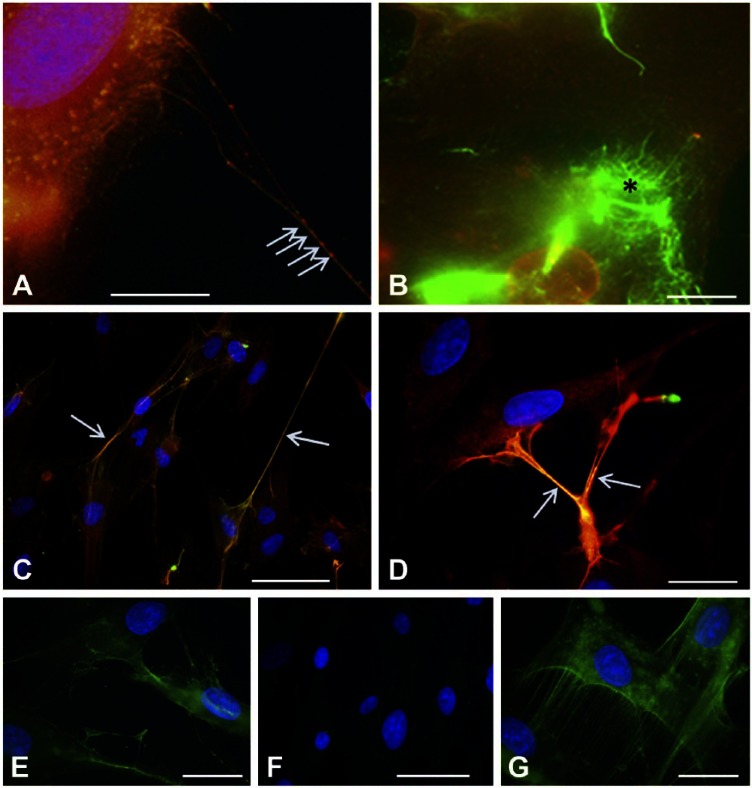
A. Cultured dermal fibroblast treated for 1 hr with recombinant human G1 (rhG1) showing localization (arrows) to hyaluronan (HA) strands. B. Fibroblasts treated for 1 hr with biotinylated HA binding protein (bHABP; 4 µg/ml), followed by treatment with rhG1 for 1 hr, followed by fixation and staining with streptavidin (green) and antibody to histidine (*His*) tag on rhG1 (red), showing absence of colocalization, indicating binding of rhG1 to HA (asterisk) is blocked. C, D. Fibroblasts cultured for 1 hr in bHABP followed by treatment with rhG1 for 6 hr in continued presence of bHABP, fixed and stained with bHABP/streptavidin and antibody to *His* tag on rhG1, showing colocalization (yellow) and cable formation (arrows). E, F. Fibroblasts treated for (E) 1 hr and (F) 7 hr, with bHABP (4 µg/ml), fixed, and stained with streptavidin (green) showing loss of bHABP binding to HA over time. G. 7-hr bHABP-treated cells fixed and stained with bHABP/streptavidin showing presence of HA cell surface strands. Scale bars A, B, 5 µm; C, 25 µm; D, E, G, 10 µm; F, 20 µm.

Binding of rhG1 to HA was apparent 1 hr after addition of rhG1 (Fig. 6A). Pretreatment of cultures for 1 hr with bHABP (4 µg/ml), followed by treatment with rhG1 for a further 1 hr, then fixation and staining with streptavidin and anti-*His*, showed an absence of colocalization (Fig. 6B); continued treatment with rhG1 out to 6 hr after the bHABP pretreatment, with the bHABP still present, and staining with bHABP/streptavidin and anti-*His*, resulted in both colocalization and cable formation (Fig. 6C, D). Fibroblasts treated with bHABP for 1 and 7 hr, prior to fixation and staining with streptavidin alone, showed that the bHABP, present at 1 hr, was lost by 7 hr (Fig. 6E, F). Subsequent staining of the 7-hr cultures with bHABP/streptavidin revealed typical cell surface HA strands (Fig. 6G). These findings are consistent with bHABP occupation of binding sites for rhG1 on HA for a limited time (at least 1 hr), and with loss over a longer period (6 hr), allowing for rhG1 binding and promotion of cable formation.

**Figure 7.**
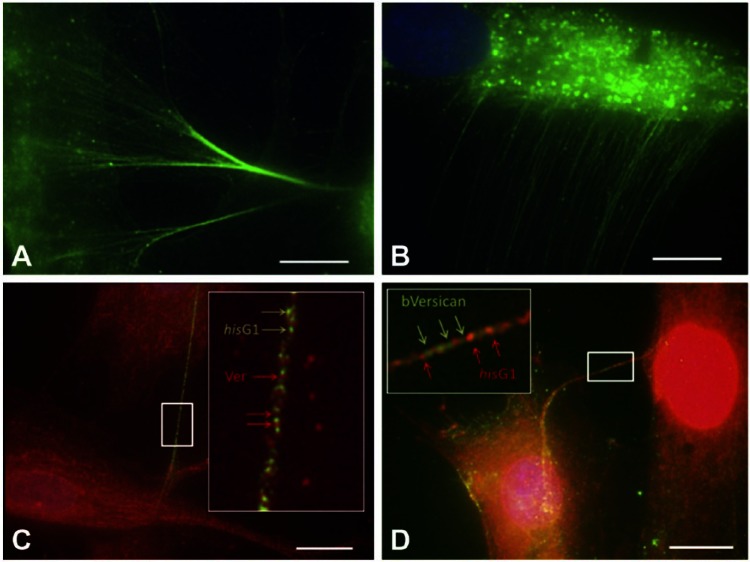
A and B. Comparison of recombinant human G1 (rhG1)–treated (A) and versican-treated (B) dermal fibroblasts, treated (10 µg/ml) for 24 hr, fixed, and stained with biotinylated hyaluronan binding protein (bHABP)/streptavidin (green), showing lack of cable formation with versican treatment, in contrast to rhG1 treatment. C. HA cable in 24 hr rhG1-treated fibroblast culture stained with antibody to histidine (*His*) tag on G1 (green) and for endogenous versican (V1) (antiversican ab177480) (red), showing both rhG1 and versican on HA strands. D. HA cable in culture treated for 22 hr with rhG1 followed by 2 hr treatment with bVersican (10 µg/ml) in continued presence of rhG1, fixed, and stained for *His* tag on G1 (red) and for bVersican with streptavidin (green), showing presence of bVersican between rhG1 deposits. Small boxed areas in C and D over cables shown as digitally magnified inserts. Scale bars, 5 mm.

In contrast to rhG1, cable formation did not occur in cultures of dermal fibroblasts treated with V0/V1 versican, at concentrations ranging from 2 to 15 µg/ml (compare Fig. 7A, B). V0/V1 versican, as demonstrated previously,^13^ locates to cell surface HA strands with a periodic distribution, and endogenous V0/V1 was present on HA cables induced by rhG1, and was located between the *His*-stained rhG1 deposits (Fig. 7C), indicating sufficient space for intact V0/V1 versican binding in addition to rhG1. Furthermore, addition of bVersican to rhG1-treated cultures, after cables had formed, showed the added bVersican also localizes between the rhG1 deposits (Fig. 7D), in the same manner as endogenous versican (Fig. 7C), again indicating sufficient space for both V0/V1 versican and rhG1 to be accommodated on HA strands.

**Figure 8.**
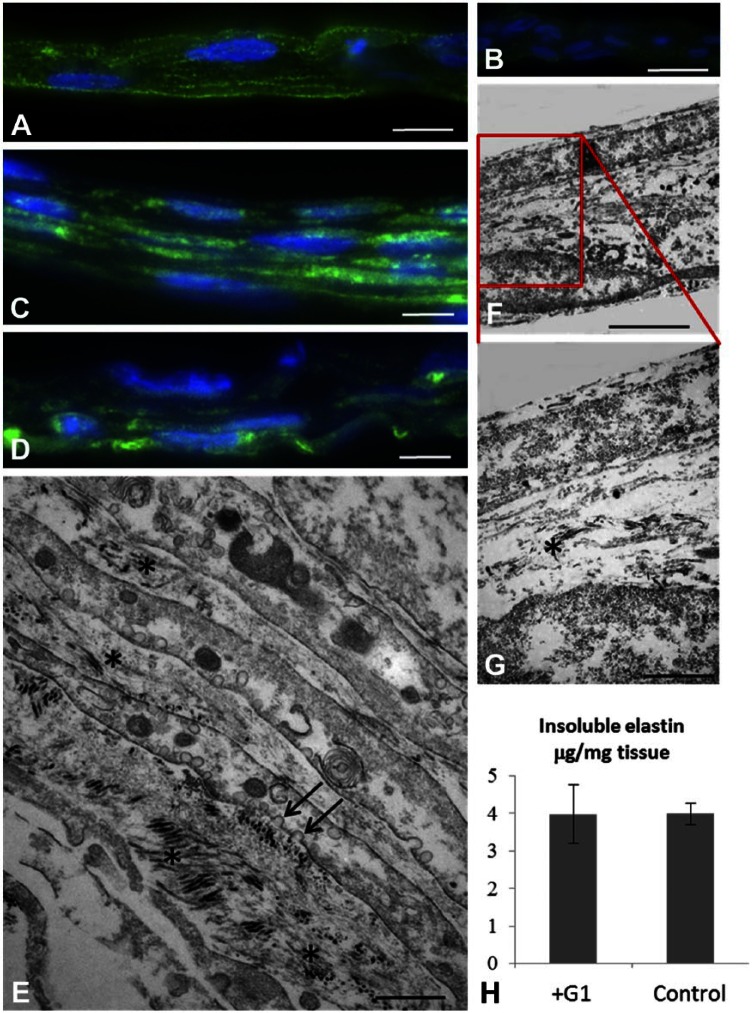
A and B. Layered dermal fibroblasts cultured for 4 weeks and (A) treated throughout with recombinant human G1 (rhG1) 10 mg/ml every 3 days in fresh medium, or (B) fresh medium alone, fixed and immunostained with antihistidine (anti-*His*; green). C and D. rhG1-treated (C) and control (D) 4-week cultures immunostained for α actin. E. Electron micrograph of 4-week rhG1-treated fibroblasts showing layered structure, deposition of collagen rich matrix (asterisks), and clearly defined pinocytotic vesicles at cell surface. F and G. Electronmicrographs of 4-week control fibroblasts showing less well-defined cell and matrix structure with scattered collagen (asterisk) in a loose matrix. H. Insoluble elastin content in rhG1-treated and control 4-week skin sheets. Error bars are SEM. Scale bars A, C, D, 10 µm; B, 20 µm; E, G, 2 µm; F, 5 µm.

Treatment of cultured fibroblasts with rhG1 for 4 weeks resulted in formation of a highly organized and layered tissue structure compared with untreated controls (Fig. 8). rhG1 localized in an array of fine punctate deposits along the surfaces of extended cells (Fig. 8A), not seen in untreated cultures (Fig. 8B). The rhG1-treated cultures stained strongly for α actin compared with control cultures where α actin was generally restricted to cells adjacent to the substrate (Fig. 8C, D). Electron microscopy of rhG1-treated cultures (Fig. 8E) showed multilayers of elongated fibroblasts in an ECM rich in collagen in which fibrils were more densely packed than in control cultures (Fig. 8F, G). Pinocytotic vesicles were also prominent in the rhG1-treated cells, but not seen in control cells which had less well-defined membranes. Small deposits of elastin and associated microfibrils were seen in the ECM and were similar in treated and control cultures, as were levels of insoluble elastin (Fig. 8H).

## Discussion

We have shown that a recombinant G1 domain of versican binds in a periodic pattern to HA strands extending from cultured dermal fibroblasts and induces aggregation of HA strands into long cables that extend from cell surfaces and frequently link adjacent cells. The aggregation of HA strands into cables was most clearly seen close to cell surfaces. At increasing distance from cell surfaces, the aggregation of strands progressively increased. In large cables with multiple strands of HA, staining for HA and for G1 overlapped, but where aggregation of just a few aggregated HA strands could be resolved, the attached G1 deposits had a periodic distribution of about 100 nm, similar to their distribution on individual strands, indicating a highly ordered structure to the cables.

The finding that rhG1 interacts with HA produced by cultured cells is consistent with previous demonstrations that a general feature of the members of Link module superfamily is their binding to HA, where multiple protein molecules can bind on a single HA chain.^17,18^ In addition, there is evidence that protein–protein interactions can cross-link HA chains,^17,19,20^ which suggests that there may be “homotypic” interaction of G1 domains mediating cable formation. A similar model for the interaction of multiple versican G1 domain-containing fragments with HA in which the fragments not only interact with HA but also mediate protein–protein interactions has been proposed.^16^

It is not clear, however, whether the rhG1-mediated aggregation of HA strands is directly responsible for cable formation and, in particular, whether the drawing together of adjacent HA strands occurs through the homotypic interaction. HA strands could also be drawn together by mechanical force. The arrangement of straight parallel HA strands within most cables suggests that the strands are under tensional force; thus, cables may be a result of HA strands being physically drawn together, possibly as cells divide then move apart over time, resulting in HA strands being drawn into cables that bridge between cells.

Aggregation due to mechanical forces, however, does not account for the distribution pattern of G1 aggregates along “fused” HA strands in which the periodicity is similar to single strands. Findings from other studies show that Link module-containing proteins can interact with each other to actively bring together adjacent HA strands, resulting in compaction or condensation of HA surface films. For example, TSG-6, with its single Link and CUB modules, is able to bind to HA and to interact with other Tumor necrosis factor-inducible gene 6 protein (TSG-6) molecules (possibly via the CUB domains) promoting collapse of the complex and rigidifying HA networks.^19^

The G1 domain of versican, however, differs from TSG-6 in two important respects; first, it has two Link modules in its HA-binding domain, and second, the G1 region has a different mode of interaction with HA compared with TSG-6, which together may lead to stabilization of distinct conformations of the polysaccharide within the HA-binding grooves of the two proteins.^21–23^ Thus, TSG-6 binding does not replicate the mechanism by which G1 coalesces HA strands, consistent with the possibility that different HA-binding proteins can mediate differential cross-linking mechanisms.

The increased diameter of G1 deposits on fused HA strands compared with single strands, without a change in periodicity pattern, suggests direct interaction between the G1 molecules. It is possible that the immunoglobulin-like region of G1 could facilitate homotypic interactions^24^ in a manner analogous to the proposed CUB interactions in TSG-6.^19^ The interaction of the versican G1 domain with HA is likely mediated via a continuous shallow binding groove that extends across the surface of the tandem Link modules;^21,25^ this can accommodate approximately a decasaccharide of HA, that is, with a length of about 5 nm.^21,26^ Thus, on this basis it would have been anticipated that many versican or G1 molecules could be bound to a 100- to 200-nm region of HA, making the periodicity seen here surprising. The larger versican variants V0 and V1, with their constituent GAG chains, however, have also been shown to localize to cell surface HA strands with a similar periodicity of 100 to 200 nm.^13^ It is possible that induction of HA cable formation requires or imposes a low level of G1 occupancy, or that there is a higher level of bound G1 (or versican) molecules than is apparent, and that not all of these are detectable, for example, due to steric constraints. We did find, using double immunofluorescence, that versican molecules, both endogenous V0/V1 and added bVersican, bind to the same strands of HA between the attached G1, confirming that there is sufficient space for accommodation of multiple HA-binding proteins over distances of ~100 nm. In the absence of the rhG1, however, V0/V1 did not induce cable formation; thus, rhG1 confers a different structural organization of the HA.

Binding of rhG1 to HA was evident as early as 1 hr after treatment and was blocked by pretreatment for 1 hr with bHABP. In the continued presence of bHABP for a further 6 hr, however, the added rhG1 again bound to and aggregated HA strands into cables, suggesting rhG1 may displace the bHABP or that new HA strands are synthesized in that time frame. Over the same time period, however, bHABP alone showed loss of binding to HA, despite the presence of cell surface HA strands. Thus, it is unlikely that rhG1 displaces bHABP. The formation of cables by 7 hr, however, and their persistence at 24 hr indicate that rhG1 binding is stable over this time period. A caveat to these and the other results of this study is that we do not know the local concentrations of rhG1 or bHABP at the cell surface and whether the observations hold for higher concentrations and longer time periods.

The rhG1-treated dermal fibroblasts grew more slowly in culture, an effect that persisted for a 2-week period following a single treatment. It is not known how rhG1 influences growth, or whether there are apoptotic effects, but notably the rhG1-induced cables were observed to tether cells to each other, an arrangement that may have inhibited both migration and the mechanics of cell division. In contrast, however, in other studies, forced expression of the versican G1 domain has been shown to enhance growth and reduce adhesion, with the latter feature the likely permissive driver of proliferation.^27^ In the same system, addition of recombinant versican G1 (expressed in bacteria) similarly enhances growth, although to a lesser degree. Full-length versican is the most effective at enhancing proliferation and reducing adhesion, both of which are moderated by removal of the G1 domain.^27^ G3, through its epidermal growth factor (EGF) domains, enhances growth but does not affect adhesion.^28^ The interplay between adhesion and proliferation may be particularly important in our system as the cables may interfere with cell detachment necessary for division to take place.

The addition of rhG1 to long-term cultures of dermal fibroblasts, over a period of 4 weeks, during which multilayers of cells formed with an intervening ECM, resulted in a significant change in morphology. The treated cells were notably elongated and layered, with a defined surface membrane with numerous pinocytotic vesicles, and a high content of α actin, characteristic of myofibroblasts and smooth muscle cells. The ECM between the cells contained clearly defined collagen fibrils, as well as elastin-associated microfibrils, packed much more densely than in control cultures in which the ECM was characterized by a loose network of components. The cells in control cultures were more loosely arranged and had poorly defined membranes. These findings suggest the rhG1 may promote differentiation, as seen in tissues in which cells express V3.^6^ Smooth muscle cells expressing V3 and seeded into ballooned vessel wall form a highly structured and layered neointima with elongated contractile cells and a compact, elastin-rich and versican-depleted matrix.^8^ Similarly, in the dermis of cultured skin sheets formed by V3-expressing fibroblasts, the elastin content is increased.^9^ The V3 variant of versican, which contains the G1 and G3 domains spliced together and no GAG chains,^29^ behaves very differently from the other variants; V3 increases synthesis and deposition of elastic fibers,^5,7–10^ promotes smooth muscle cell differentiation,^6^ and also dampens inflammatory responses.^6,11^ It is not clear whether these effects are properties of the G1 domain, the G3 domain, or both. Our present findings indicate that the G1 domain alone may be sufficient to induce these latter changes, although not (under conditions used here) an elastin response. There are, of course, caveats to comparing G1 and V3. Findings for V3 come from cells undergoing forced expression of V3, whereas the G1 data presented here are from cells exposed to exogenously added rhG1, where the amounts of endogenous HA-binding proteins, and variations in culture conditions, are factors that may influence structural organization of matrix around cells.

Finally, the formation of HA cable-like structures is of significance because similar structures have been generated by stromal cells in response to agonists that induce endoplasmic reticulum stress and cause HA accumulation.^12,30–35^ Furthermore, these cables have been identified as ligands for leukocyte adhesion and proposed to have both pro- and anti-inflammatory activities.^12,13,17,30,32,36–39^ The ability of HA to function as either a pro- or anti-inflammatory molecule is dependent on a number of factors, including its size, the microenvironment, localization, and availability of specific binding partners.^17,40^ Whether the cables generated by rhG1 treatment of dermal fibroblasts possess leukocyte adhesive properties awaits further experimentation.

The present study, however, demonstrates that the G1 domain of versican has the potential to significantly affect the organization of the ECM, and our findings provide new insights into the diverse functions of HABPs and their isoforms.
